# Augmenting anti-CD19 and anti-CD22 CAR T-cell function using PD-1-CD28 checkpoint fusion proteins

**DOI:** 10.1038/s41408-021-00499-z

**Published:** 2021-06-04

**Authors:** Franziska Blaeschke, Dana Stenger, Antonia Apfelbeck, Bruno L. Cadilha, Mohamed-Reda Benmebarek, Jasmin Mahdawi, Eva Ortner, Mareike Lepenies, Nicola Habjan, Felicitas Rataj, Semjon Willier, Theresa Kaeuferle, Robbie G. Majzner, Dirk H. Busch, Sebastian Kobold, Tobias Feuchtinger

**Affiliations:** 1 Department of Pediatric Hematology, Oncology and Stem Cell Transplantation, Dr. von Hauner Children’s Hospital, University Hospital, LMU Munich, Munich, Germany; 2 German Cancer Consortium (DKTK), Partner Site Munich, Munich, Germany; 3 Center for Integrated Protein Science Munich (CIPSM) and Division of Clinical Pharmacology, Department of Medicine IV, University Hospital, LMU Munich, Munich, Germany; 4 National Center for Infection Research (DZIF), Munich, Germany; 5 Department of Pediatrics, Stanford University School of Medicine, Stanford, CA USA; 6 Stanford Cancer Institute, Stanford University School of Medicine, Stanford, CA USA; 7 Institute for Medical Microbiology, Immunology and Hygiene, Technical University Munich, Munich, Germany; 8 Focus Group “Clinical Cell Processing and Purification”, Institute for Advanced Study, TUM, Munich, Germany

**Keywords:** Acute lymphocytic leukaemia, Acute lymphocytic leukaemia, Immunotherapy

Dear Editor,

Despite high initial responses after treatment with anti-CD19 chimeric antigen receptor (CAR) T cells in pediatric B-cell precursor acute lymphoblastic leukemia (BCP-ALL), 40–50% of patients relapse within 24 months^1,2^. In solid tumors, PD-1/-L1 (programmed death (ligand) 1) blockade enhances, i.e., Her2-specific CAR function^3^. Although BCP-ALL and bone marrow T cells express PD-L1/PD-1^4,5^, clinical benefit of PD-1/PD-L1-blocking antibodies is low and this mechanism in BCP-ALL remains controversial. Furthermore, PD-1/PD-L1-blocking antibodies trigger autoimmune side effects by uncontrolled T-cell proliferation of auto-reactive T cells. An attractive targeted alternative are synthetic fusion proteins—receptors with extracellular and transmembrane domains of PD-1 that are fused to the intracellular domain of CD28 and thus turn PD-1-mediated inhibitory signals into CD28-mediated T-cell stimulation. Here, we systematically characterize a fully human PD-1-CD28 fusion protein in combination with anti-CD19 and anti-CD22 CAR T cells. Aim of this study is to create an adaptable system to specifically increase functionality of anti-leukemia CAR T cells in order to protect CAR T cells from leukemia-induced inhibition.

To identify the impact of PD-L1/PD-1 inhibition in BCP-ALL, upregulation of PD-L1 was analyzed on leukemic blasts (cell line and bone marrow blasts of pediatric BCP-ALL patients) in response to Th1 cytokines Interferon-gamma (IFN-γ) and tumor necrosis factor alpha (TNF-α) (Fig. 1A, B). Primary blasts showed an interindividual heterogenous response with upregulation, downregulation or nonresponding patient samples. To verify whether PD-L1 expression on ALL cells inhibits T-cell responses, second-generation anti-CD19 CAR T cells (19_BB_3z) were co-cultured with CD19^+^ and CD19^+^/PD-L1^+^ target cells. Twenty-four hours later, CAR T cells co-cultured with PD-L1^+^ targets showed decreased levels of Th1 cytokine secretion (Fig. 1C, D). These data show that PD-1/PD-L1 can mediate T-cell inhibition after/during T-cell response against BCP-ALL.

**Fig. 1:**
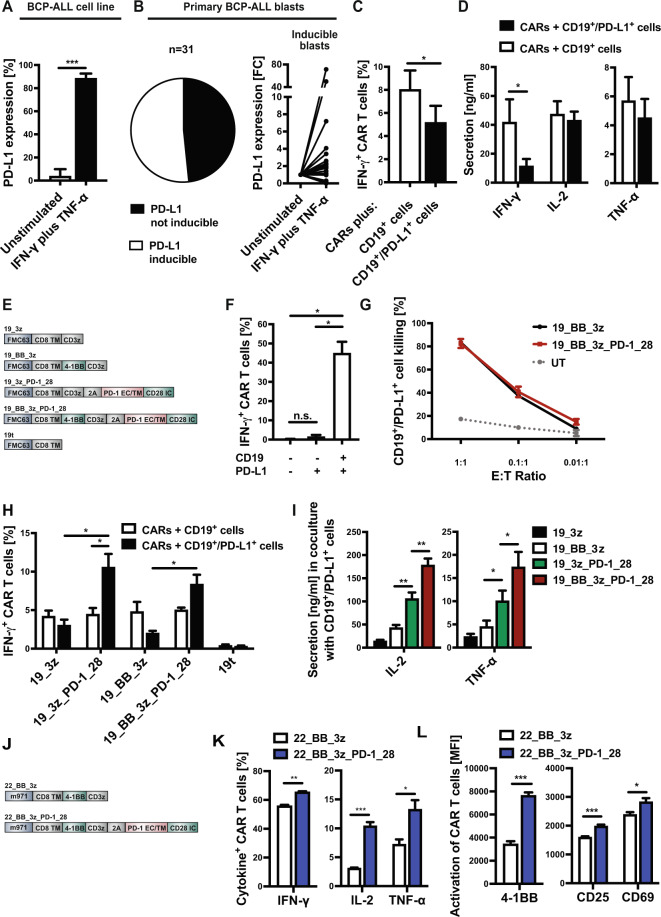
Impact of PD-L1 interaction on T-cell functionality and characterization of anti-CD19 and anti-CD22 CAR T cells with PD-1-CD28 fusion protein. **A** PD-L1 expression on Nalm-16 cells was analyzed 24 h after stimulation with IFN-γ and TNF-α. **B** Primary ALL blasts from 31 different pediatric BCP-ALL patients were stimulated with IFN-γ and TNF-α for 24 h. Pie chart shows percentage of PD-L1-inducible vs. PD-L1-non-inducible samples (left panel). Surface PD-L1 expression in inducible patients (*n* = 16) is shown in the right panel (fold-change of stimulated sample vs. unstimulated sample). **C** Conventional second-generation CAR T cells were co-cultured with CD19^+^ and CD19^+^/PD-L1^+^ Daudi cells. Percentage of intracellular IFN-γ^+^ in CAR T cells was analyzed 24 h later. **D** 19_BB_3z T cells were co-cultured with Daudi cells for 24 h and co-culture supernatant was analyzed for concentration of IFN-γ, IL-2 and TNF-α in a flow-cytometry-based assay. **E** Schematic illustration of CAR constructs and negative control (19t). **F** 19_3z_PD-1_28 CAR T cells were co-cultured with CD19^-^/PD-L1^-^, CD19^-^/PD-L1^+^ and CD19^+^/PD-L1^+^ target cells (transduced K562 cells). Intracellular cytokine stain was performed 24 h later and showed no unspecific activation of the PD-1-CD28 fusion protein. **G** Cytotoxicity of conventional second-generation CAR T cells (19_BB_3z) and second-generation CAR T cells with fusion protein (19_BB_3z_PD-1_28) was analyzed 48 h after co-culture with CD19^+^/PD-L1^+^ K562 cells. **H** CAR T cells were co-cultured with CD19^+^ and CD19^+^/PD-L1^+^ Daudi cells. Intracellular stain for IFN-γ was performed 24 h later. **I** CAR T cells were co-cultured with CD19^+^/PD-L1^+^ target cells and concentration of IL-2 and TNF-α was analyzed in the supernatant 24 h after start of co-culture. **J** Schematic illustration of the second-generation anti-CD22 CAR and the version with fusion protein used in this study. **K** Intracellular cytokine stains of anti-CD22 CAR T cells 24 h after co-culture with Daudi cells showed increased cytokine release of PD-1-CD28 CAR T cells. **L** Activation markers 4-1BB, CD25 and CD69 were increased in PD-1-CD28 CAR T cells over conventional anti-CD22 CAR T cells 24 h after co-culture with Nalm-6 cells. *N* ≥ 3 individual donors (**C**, **D**, **F**, **G**, **H** and **I**). *N* ≥ 2 individual donors (**K** and **L**). Statistical significance was calculated using *t*-test. UT untransduced T cells, E:T ratioeffector to target ratio, FC fold-change, BCP-ALL B-cell precursor ALL, n.s. not significant, MFI geometric mean fluorescent intensity.

To circumvent inhibition through PD-1/PD-L1, CAR T cells with PD-1-CD28 fusion proteins were generated. The PD-1-CD28 fusion protein is designed to transform inhibitory signals of leukemic cells (PD-1) into T-cell stimulation (CD28). Conventional first/second-generation CAR T cells were generated (19_3z, 19_BB_3z) and extended by the fusion protein linked via a 2A sequence (19_3z_PD-1_28, 19_BB_3z_PD-1_28) (Fig. 1E). A CAR construct lacking stimulatory domains served as negative control (19t). CAR T cells with PD-1-CD28 fusion protein showed transduction rates >60% and strong correlation between CAR (myc tag) and PD-1 expression (Supplementary Fig. 1A, B). Mean expansion rate was >100-fold for all CAR constructs (Supplementary Fig. 1C). To exclude unspecific activity of the PD-1-CD28 fusion protein in absence of the CAR signal, K562 cells were transduced with either only PD-L1, the combination of CD19/PD-L1 or left untransduced. 24 h after start of co-culture, 19_3z_PD-1_28 cells showed IFN-γ release only when both the CAR (CD19) and the fusion protein target (PD-L1) were present (Fig. 1F). No increase in IFN-γ release was seen in the absence of the CD19-mediated CAR signal. Cytotoxicity, proliferation, and cytokine release assays confirmed strong CD19-specific functionality of PD-1-CD28 CARs (Supplementary Fig. 2A–G). 19_3z_PD-1_28 cells specifically upregulated activation markers and differentiated from mostly central memory T cells to >95% effector memory T cells after co-culture with CD19^+^ targets (Supplementary Fig. 2H, I). Background expression levels of co-inhibitory molecules were low except for PD-1 proving successful transduction with the fusion protein (Supplementary Fig. 2J, K).

We next compared PD-1-CD28 CARs with conventional anti-CD19 CARs and co-cultured them with PD-L1^+^ or PD-L1^−^ cell lines with similar CD19 levels (Supplementary Fig. 3A). While no difference in short-term cytotoxicity of PD-1-CD28 CARs vs. conventional CARs was observed (Fig. 1G and Supplementary Fig. 3B), PD-1-CD28 CAR T cells outcompeted conventional CARs in regard to IFN-γ release in the presence of PD-L1 (Fig. 1H). Next, IL-2 and TNF-α concentrations were analyzed in the supernatant of CARs co-cultured with CD19^+^/PD-L1^+^ cell lines (Fig. 1I). Again, PD-1-CD28 CAR T cells showed higher secretion of IL-2 and TNF-α proving that the fusion protein can add functionality in the presence of PD-L1.

To verify that this system is adaptable to other CAR specificities, a second-generation anti-CD22 CAR (m971) with PD-1-CD28 fusion protein was generated (Fig. 1J). Flow cytometric analysis showed high transduction rates and strong correlation between CD22 CAR and PD-1 expression in PD-1-CD28 CARs (Supplementary Fig. 4A, B). Intracellular cytokine stains for IFN-γ, IL-2, and TNF-α confirmed superior functionality of PD-1-CD28 CD22 CARs in the presence of PD-L1 (Fig. 1K). In addition, anti-CD22 CARs showed increased expression of activation markers after co-culture with PD-L1^+^ target cells (Fig. 1L). In summary, PD-1-CD28 fusion proteins can add anti-leukemic functionality to conventional CAR T cells and can be combined with CARs of different specificity.

Next, to better mimic exhaustion/multiple antigen encounter, target cells were added to anti-CD19 CARs every 3 to 4 days and target-cell killing was analyzed. Whereas conventional first- and second-generation CARs lost cytotoxic capacity over time, PD-1-CD28 CAR T cells were able to kill about 80% of the freshly added target cells even after multiple antigen encounter (Fig. 2A). After first as well as after fourth stimulation with target cells, PD-1-CD28 CAR T cells showed increased expression of IFN-γ and TNF-α compared to conventional CARs (Fig. 2B) confirming their improved fitness even after multiple stimulations with targets. Second donor shown in Supplementary Fig. 5.

**Fig. 2:**
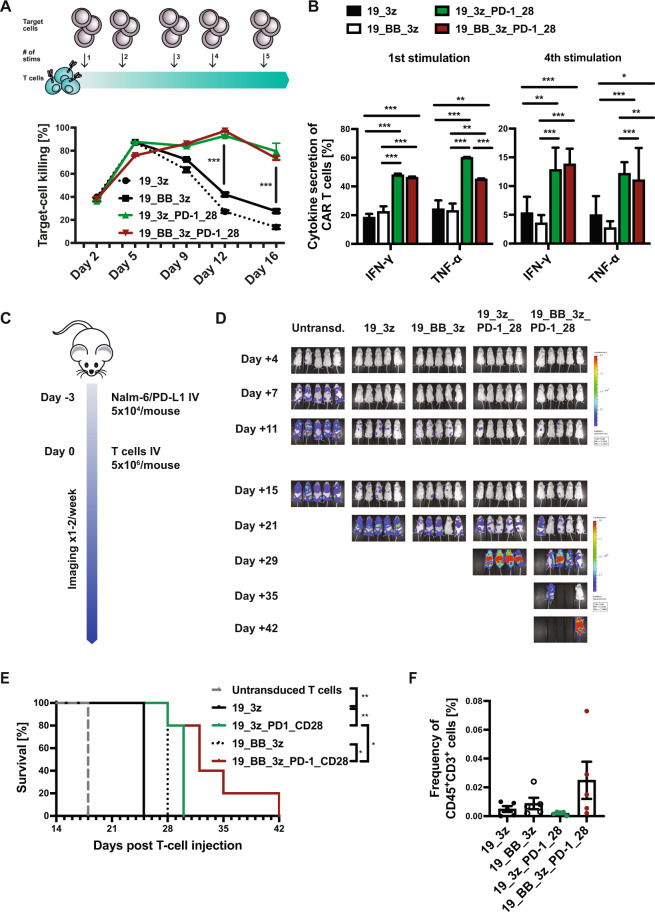
Functionality of PD-1-CD28 CAR T cells after multiple antigen encounter and in vivo. **A** Schematic illustration and cytotoxicity curves of the multiple stimulation assay. T cells were stimulated with fresh target cells (Daudi) every 3 to 4 days. Killing of freshly added target cells at every re-stimulation timepoint was determined by flow-cytometry-based cytotoxicity assay. Exemplary plot for one donor shown, second donor is shown in Supplementary Fig. 5. **B** Intracellular cytokine stain of CAR T cells was performed 24 h after first and 24 h after fourth re-stimulation. **C** Schematic illustration of the leukemia model used to evaluate in vivo functionality. 5 × 10^6^ CAR T cells were injected 3 days after injection of 5 × 10^4^ Nalm-6 cells with PD-L1 overexpression. **D** Bioluminescence imaging was performed once to twice per week until day + 39 after T-cell injection. Days after T-cell injection are shown. **E** Survival analysis of mice, which received untransduced T cells, conventional first- and second-generation CAR T cells or CAR T cells with fusion protein. **F** Frequency of CAR^+^CD45^+^CD3^+^ (CAR) T cells was analyzed by flow-cytometry on day + 22 after T-cell injection. *N* = 5 mice per group. Statistical significance was calculated using *t*-test (**A**, **B**) or Log-rank (Mantel-Cox) test (**E**).

Finally, the functional relevance of PD-L1 expression was analyzed in vivo. First, PD-L1-transduced Nalm-6 cells were injected head-to-head with conventional PD-L1^-^ Nalm-6 cells into NSG mice. In line with the in vitro findings, PD-L1 overexpression resulted in accelerated leukemia progression/reduced survival compared to conventional Nalm-6 (Supplementary Fig. 6A). To test functionality of PD-1-CD28 anti-CD19 CAR T cells in vivo, a dose of 5 × 10^4^ PD-L1^+^ Nalm-6 leukemia cells was injected followed by IV injection of 5 × 10^6^ T cells 3 days later (Fig. 2C). The leukemia dose was reduced in order to (1) show an effect of the conventional CARs over control treatment and (2) have enough bandwidth to illustrate the improved effect of PD-1-CD28 CARs before the mice eventually succumb to the more aggressive PD-L1^+^ leukemia. Bioluminescence imaging showed higher leukemia signal in mice treated with conventional CARs vs. PD-1-CD28 CARs (Fig. 2D and Supplementary Fig. 6B). PD-1-CD28 CAR T cells improved survival over conventional CAR T cells (Fig. 2E). Twenty-two days after T-cell injection, second generation CAR T cells with fusion protein trended towards lower percentage of leukemic cells (Supplementary Fig. 6C) and higher frequencies of CAR T cells (Fig. 2F) in the peripheral blood compared to conventional second-generation CAR T cells. No increased toxicity of fusion receptor CAR T cells was observed.

In conclusion, PD-1-CD28 CAR T cells can outcompete conventional CAR T cells in the presence of PD-L1 both, in vitro and in vivo. Here, we used a synthetic fully human PD-1-CD28 fusion protein to ensure low immunogenicity compared to previously reported murine constructs^6,7^. Efficacy of murine PD-1-CD28 fusion proteins was described in combination with tumor-specific T-cell receptors^6,7^ in pancreatic cancer and Non-Hodgkin Lymphoma models. Xiaojun Liu^8^ and Hui Liu^9^ described human PD-1-CD28 fusion proteins using shorter parts of PD-1 (AA1-155) and larger parts of CD28 (AA141-220) and reached increased functionality in diffuse large B-cell lymphoma. To our knowledge, PD-1-CD28 fusion proteins have not been analyzed in combination with anti-CD22 CAR T cells yet. Here, we use a fusion protein with a longer PD-1 (AA1-191) and a shorter CD28 portion (AA180–220) as the murine counterpart of this receptor has been shown to be superior to other PD-1-CD28 designs in an OT-1 model^6^. We did not observe toxicity in our in vivo model. However, a suicide switch could be easily integrated into the multicistronic construct^10^. Moreover, as an ultima ratio, steroids could be administered in case of overstimulation and toxicity of PD-1-CD28 CAR T cells.

Recently, CAR T cells targeting the PD-1 axis, i.e., through PD-1 (*PDCD1)* knockout (KO), dominant-negative PD-1 receptors or antibody secretion showed increased functionality^11–13^. However, recent studies highlighted that *PDCD1* is a master gene suppressing oncogenic T-cell signaling and *PDCD1* deletions are recurrently observed in T-cell lymphomas^14^ hinting at a potential risk of *PDCD1* KO. Thus, the concept of PD-1-CD28 fusion proteins is attractive as (1) it does not require KO of the endogenous gene, (2) the fusion protein only signals when CAR signaling is present, (3) no repeated administration of a drug is necessary (4) it is easily adaptable to a variety of different CAR specificities, and (5) in contrast to dominant-negative receptors, this design increases cytokine release even beyond the natural level of cytokine secretion in the absence of PD-L1.

The relevance of the PD-1/PD-L1 axis in ALL remains under investigation. We found that pediatric ALL blasts can upregulate PD-L1 with an interindividual heterogeneous expression pattern. This is in line with recent studies highlighting strong spatial/temporal heterogeneity of PD-L1 expression in malignant tumors^15^. Our in vitro data show that activation through the CAR itself is not impaired in the absence of PD-L1, but CAR function can be augmented beyond levels of conventional CARs in the presence of PD-L1. Future clinical studies will evaluate for each individual patient, whether anti-CD19 or anti-CD22 CARs with PD-1-CD28 fusion proteins can improve conventional CAR functionality even in the absence of tumor/leukemia PD-L1 expression in patients.

## Supplementary information

Supplement

## References

[1] Gardner RA (2017). Intent-to-treat leukemia remission by CD19 CAR T cells of defined formulation and dose in children and young adults. Blood.

[2] Maude SL (2018). Tisagenlecleucel in children and young adults with B-cell lymphoblastic leukemia. N. Engl J. Med..

[3] John LB (2013). Anti-PD-1 antibody therapy potently enhances the eradication of established tumors by gene-modified T cells. Clin. Cancer Res..

[4] Feucht J (2016). T-cell responses against CD19+ pediatric acute lymphoblastic leukemia mediated by bispecific T-cell engager (BiTE) are regulated contrarily by PD-L1 and CD80/CD86 on leukemic blasts. Oncotarget.

[5] Blaeschke F (2020). Leukemia-induced dysfunctional TIM-3(+)CD4(+) bone marrow T cells increase risk of relapse in pediatric B-precursor ALL patients. Leukemia.

[6] Kobold, S. et al. Impact of a new fusion receptor on PD-1-mediated immunosuppression in adoptive T cell therapy. *J. Natl Cancer Inst*. **107**, djv146 (2015). 10.1093/jnci/djv146 PMC4609553 26105028

[7] Rataj F (2018). PD1-CD28 fusion protein enables CD4+ T cell help for adoptive T cell therapy in models of pancreatic cancer and non-hodgkin lymphoma. Front. Immunol..

[8] Liu X (2016). A chimeric switch-receptor targeting PD1 augments the efficacy of second-generation CAR T cells in advanced solid tumors. Cancer Res..

[9] Liu H (2020). CD19-specific CAR T cells that express a PD-1/CD28 chimeric switch-receptor are effective in patients with PD-L1-positive B-cell lymphoma. Clin. Cancer Res..

[10] Straathof KC (2005). An inducible caspase 9 safety switch for T-cell therapy. Blood.

[11] Li S (2017). Enhanced cancer immunotherapy by chimeric antigen receptor-modified T cells engineered to secrete checkpoint inhibitors. Clin. Cancer Res..

[12] Rafiq S (2018). Targeted delivery of a PD-1-blocking scFv by CAR-T cells enhances anti-tumor efficacy in vivo. Nat. Biotechnol..

[13] Rupp LJ (2017). CRISPR/Cas9-mediated PD-1 disruption enhances anti-tumor efficacy of human chimeric antigen receptor T cells. Sci. Rep..

[14] Wartewig T (2017). PD-1 is a haploinsufficient suppressor of T cell lymphomagenesis. Nature.

[15] Zhou KI (2020). Spatial and temporal heterogeneity of PD-L1 expression and tumor mutational burden in gastroesophageal adenocarcinoma at baseline diagnosis and after chemotherapy. Clin. Cancer Res..

