# Supplementary material for: Augmenting anti-CD19 and anti-CD22 CAR T-cell function using PD-1-CD28 checkpoint fusion proteins
Source: Blood Cancer J. 2021 Jun 4;11(6):108. doi: 10.1038/s41408-021-00499-z (PMC8178409; doi:10.1038/s41408-021-00499-z)

Materials and Methods

Patients and samples

This study was approved by the Institutional Ethical Review Board ("Ethikkommission bei der LMU München"), approval number 435-15/ 17-163. The study was performed in accordance with the Declaration of Helsinki. Patients/ their representatives and healthy donors gave written informed consent. All animal trials were performed in accordance with the current ethical standards of the official committee on animal experimentation (written approval by Regierung von Oberbayern).

Stimulation of cell lines and primary ALL cells

Primary bone marrow ALL cells and Nalm-16 cells were stimulated with 100 ng/ml IFN-γ (Imukin, Boehringer Ingelheim, Ingelheim, Germany) and 100 ng/ml TNF-α (Cellgenix, Freiburg, Germany). After 48 hours, PD-L1 surface expression was determined by flow cytometric staining for PD-L1-BV421 (Biolegend, San Diego, California, USA).

Transduction of cell lines

K562 cell line was transduced with human CD19, human PD-L1 or the combination of both. Daudi cell line was transduced with human PD-L1. Therefore, human CD19 (NM_001770.5) and PD-L1 (AAI13735.1) were cloned into the retroviral vector pMP71 kindly provided by Christopher Baum, Hannover Medical School, Hannover, Germany. Stable producer cell lines were generated as described above and used for stable transduction of K562 and Daudi cell lines. Transduced cell lines were sorted for PD-L1-BV421 (Biolegend, San Diego, California, USA) and/ or CD19-BB515 (Becton, Dickinson and Company, Franklin Lakes, New Jersey, USA) on a BD FACS ARIA III (Becton, Dickinson and Company, Franklin Lakes, New Jersey, USA) and routinely tested for transgene expression and CD19 stability.

CAR T-cell generation

CAR and control inserts were generated by PCR cloning. Schematic representation of the constructs is shown in Fig. 1D. All constructs included a myc tag to allow detection by flow cytometry. Anti-CD19 scFv was designed in analogy to patent US20170107286 by Kochenderfer and colleagues (FMC63). Anti-CD22 scFv sequence was kindly provided by Robbie Majzner, Stanford University School of Medicine, USA. Inserts were cloned into retroviral vector pMP71 kindly provided by Christopher Baum, Hannover Medical School, Hannover, Germany. Producer cell lines (293Vec-Galv and 293Vec-RD114) were

generated for all constructs as previously published (27, 28). 293Vec-Galv and -RD114 cell lines were kindly provided by Manuel Caruso, BioVec Pharma, Montreal, Canada. Supernatant of 293Vec-RD114 cells was harvested, filtered (0.45 µm) and stored at -80 °C. Correct transduction of producer cells was verified by PCR using Q5 High-Fidelity DNA Polymerase according to the supplier's information (New England Biolabs, Ipswich, Massachusetts, USA) and subsequent Sanger sequencing (Eurofins Genomics, Ebersberg, Germany). Peripheral blood mononuclear cells (PBMCs) of healthy donors were generated via density gradient centrifugation according to the manufacturer's information (Biocoll, Biochrom, Berlin, Germany). T cells were isolated using CD4 and CD8 MicroBeads (Miltenyi Biotec, Bergisch Gladbach, Germany) and activated via T Cell TransAct, human according to the manufacturer's information (Miltenyi Biotec, Bergisch Gladbach, Germany). T cells were cultured in TexMACS GMP medium/ 2.5% human AB serum + 12.5 ng/ml interleukins 7 and 15 (Miltenyi Biotec, Bergisch Gladbach, Germany). 48 after activation, T cells were washed and transduced with the respective CAR constructs. Therefore, 24-well plates were coated with 2.5 µg RetroNectin Reagent (Takara Bio, Kusatsu, Japan) overnight at 4 °C, blocked with 2% Albumin Fraction V in PBS (Biotest, Dreieich, Germany) and washed with a 1:40 dilution of HEPES 1M (Biochrom, Berlin, Germany) in PBS (Thermo Fisher Scientific, Waltham, Massachusetts, USA). 1 ml viral supernatant was centrifuged on coated wells (3000 g, 90 minutes, 32 °C). Supernatants were discarded and $1x10^6$ T cells in 1 ml TexMACS GMP medium/ 2.5% human AB serum + 12.5 ng/ml interleukin 7 and 15 were added per well. Plates were centrifuged at 450 g, 10 minutes, 32 °C. 48 hours after transduction, T cells were washed to remove remaining virus. On day 7-12 after transduction, cellular composition and transduction rate were analysed by flow cytometry using CD3-PE-Vio770, CD4-VioGreen, CD8-APC-Vio770, CD56-APC, c-myc-FITC (Miltenyi Biotec, Bergisch Gladbach, Germany) and PD-1-BV421 (Biolegend, San Diego, California, USA). To detect the anti-CD22 CAR, Recombinant Human Siglec-2/CD22 Fc Chimera Protein in combination with Human IgG Fc APC-conjugated Antibody was used (both Research And Diagnostic Systems, Minneapolis, Minnesota, USA).

Cytotoxicity assay

NK (natural killer) / NKT cells were depleted from the CAR product using CD56 MicroBeads according to the supplier's information (Miltenyi Biotec, Bergisch Gladbach, Germany) to reduce background signal. Transduction rate was evaluated and all conditions were adjusted to the lowest transduction rate within the donor by adding untransduced cells. $CD19^+$, $PD\text{-}L1^+$, $CD19^+/PD\text{-}L1^+$ or Wildtype ($CD19^-$/PD-

L1$^-$) K562 cells were used as target cells. Target cells were labelled with CellTrace Violet Cell Proliferation Kit according to the supplier's information (Thermo Fisher Scientific, Waltham, Massachusetts, USA). CARs were co-cultured with target cells for 48 hours at various effector to target ratios (calculated on CAR$^+$ cells). Absolute count of remaining CellTrace Violet-positive cells was calculated using the MACSQuant Analyzer 10 (Miltenyi Biotec, Bergisch Gladbach, Germany) and set into relation to the count of CellTrace Violet-positive cells of control wells (target cells only).

Proliferation assay

All conditions were adjusted to the lowest transduction rate within the donor by adding untransduced cells. T cells were labeled according to CellTrace Violet Cell Proliferation Kit (Thermo Fisher Scientific, Waltham, Massachusetts, USA) and co-cultured with CD19$^+$ or Wildtype (CD19$^-$) K562 cells (E:T ratio 5:1). Cell proliferation was measured on a MACSQuant Analyzer 10 (Miltenyi Biotec, Bergisch Gladbach, Germany) before and 72 hours after stimulation. E:T ratio was calculated on the total T-cell count. For analysis, co-cultures were additionally stained for CD19-PE-Vio770, CD8-APC-Vio770, CD95-APC, c-myc-FITC (Miltenyi Biotec, Bergisch Gladbach, Germany), CD62L-APC/Cy7 and CD45RO-PE (Biolegend, San Diego, California, USA).

Intracellular cytokine stain (ICS)

All conditions were adjusted to the lowest transduction rate within the donor by adding untransduced cells. T cells were co-cultured with K562 or Daudi cells for 24 hours (E:T ratio 5:1). E:T ratio was calculated on the total T-cell count. 2 hours after stimulation, 10 µg/ml Brefeldin A (Sigma-Aldrich, Steinheim, Germany) was added. Stimulation was stopped with cold PBS 4 hours later. Intracellular stain with IFN-γ-PE (Becton, Dickinson and Company, Franklin Lakes, New Jersey, USA) and TNF-α-Pacific Blue (Biolegend, San Diego, California, USA) was performed using FIX & PERM cell Fixation & Permeabilization Kit (Thermo Fisher Scientific, Waltham, Massachusetts, USA) according to the supplier's information. Extracellular staining for CD3-PE-Vio770, CD4-VioGreen, CD8-APC-Vio770, c-myc-FITC (Miltenyi Biotec, Bergisch Gladbach, Germany) and CD56-APC (Becton, Dickinson and Company, Franklin Lakes, New Jersey, USA) was performed.

Evaluation of T-cell activation and T-cell phenotype

Expression of T-cell activation markers, phenotypic markers and co-inhibitory molecules on T cells was determined by flow cytometry prior to and after 24 and 48 hours of co-culture with target cell lines (K562, E:T ratio 5:1). Effector count was calculated on the total T-cell count. All conditions were adjusted to the lowest transduction rate within the donor by adding untransduced cells. Panels included 7-AAD, LAG-3-PE, CD62L-APC-Cy7, 2B4-APC, CD95-BV785, PD-1-BV650 4-1BB-BV421, BTLA-BV650, CTLA-4-BV421 (Biolegend, San Diego, California, USA), c-myc-FITC, CD45RO-PE-Vio770 (Miltenyi Biotec, Bergisch Gladbach, Germany), CD56-BUV737, CD8-BUV496, CD3-BUV395 CD69-BV650 (Becton, Dickinson and Company, Franklin Lakes, New Jersey, USA), TIGIT-PE (eBioscience/ Thermo Fisher Scientific, Waltham, Massachusetts, USA). Measurement was performed on a BD LSRFortessa cell analyzer (Becton, Dickinson and Company, Franklin Lakes, New Jersey, USA).

Multiplex cytokine assay

CAR T cells were co-cultured with target cells (Daudi) at an E:T ratio of 1:2. Effector-cell count was calculated based on transduction rate (CAR$^+$ cells). Supernatant of co-cultures was harvested after 24 hours and frozen at -20°C. For analysis, supernatant was thawed and diluted 1:50 with buffer using the LEGENDplex Human CD8/NK panel 13-plex (Biolegend, San Diego, California, USA). Flow-cytometric measurement was performed according to the supplier's information.

Multiple stimulation assay

T cells were thawed and rested overnight. NK (natural killer) / NKT cells were depleted from the CAR product using CD56 MicroBeads according to the supplier's information (Miltenyi Biotec, Bergisch Gladbach, Germany). T cells were incubated with PD-L1$^+$ Daudi cells at an E:T ratio of 0.2:1 (cytotoxicity assays) or 1:1 (ICS). Every three to four days T cells were re-stimulated with CellTrace Violet labelled Daudi cells (x5 total). Killing capacity was evaluated by counting living CellTrace Violet positive cells 24 hours after each re-stimulation. Intracellular cytokine stain was performed 24 hours after 1st and 4th stimulation as described above.

***In vivo* model**

All animal trials were performed in accordance with the current ethical standards of the official committee

on animal experimentation (written approval by Regierung von Oberbayern). NSG mice (NOD-scid IL2Rgamma^{null}) were maintained under specific pathogen-free conditions, had free access to food and water, and were housed with a 12h light/dark cycle and constant temperature. Nalm-6 cells were genetically modified by lentiviral transduction to express enhanced firefly luciferase and eGFP as selection marker. To compare PD-L1 transduced with conventional Nalm-6 cells, $1x10^5$ cells were injected IV. For further experiments, $5x10^4$ luciferase-positive PD-L1-transduced Nalm-6 cells were injected IV followed by $5x10^6$ T cells IV three days later. Bioluminescence imaging was performed once to twice per week. Peripheral blood was analyzed regularly to detect human leukemic cells and CAR T cells. Clinical condition and weight were evaluated regularly to detect potential toxicity of CAR T cells. Mice were sacrificed once the endpoint criteria (scoring sheet, 20% weight loss) were met.

Cell lines

Cell lines were routinely tested for identity by short-tandem repeat analyses (DSMZ, Braunschweig, Germany).

Statistics

Statistical analyses were performed using Graphpad Prism 7. All graphs represent mean plus standard error mean unless otherwise stated. * means $p<0.05$, **means $p <0.01$, *** means $p<0.001$, **** means $p<0.0001$.

Figure Legends for Supplementary Figures

Supplementary Figure 1: T-cell transduction and expansion of CARs. (A) Transduction rates were analyzed 12 days after activation by flow cytometric c-myc staining. (B) Exemplary flow cytometry plots illustrating CAR transduction in conventional CARs and simultaneous transduction of CAR and PD-1-CD28 fusion protein in PD-1-CD28 CARs. (C) Expansion rates of different CAR constructs after CD3-/CD28-based activation on day 0. N≥3 individual donors (A and C).

Supplementary Figure 2: CD19-specific functionality of PD-1-CD28 CAR T cells. (A) 19_3z_PD-1_28 CAR T cells were co-cultured with $CD19^+$ and WT ($CD19^-$) K562 cells at various E:T ratios. Killing capacity after 48 hours is shown. (B) Proliferation of 19_3z_PD-1_28 CAR T cells was analyzed 72h after co-culture with $CD19^+$ and WT ($CD19^-$) K562 cells. T cells without targets served as control. (C) Subpopulations of proliferating T cells were analyzed by flow cytometry. (D-G) Intracellular cytokine stain for IFN-γ (D, E) and TNF-α (F, G) was performed 24 hours after co-culture of 19_3z_PD-1_28 CARs with $CD19^+$ vs. WT K562 cells. E and G show IFN-γ-/TNF-α production of CAR^+ vs CAR^- T cells. (H) Expression of activation markers 4-1BB and CD69 on 19_3z_PD-1_28 CARs was analyzed by flow cytometry 24 hours after co-culture with $CD19^+$ K562 cells. (I) T-cell phenotype was determined by flow cytometric staining for CD62L, CD45RO and CD95 before and after co-culture of 19_3z_PD-1_28 CARs with $CD19^+$ K562 cells. (J) Surface expression of co-inhibitory molecules was analyzed on 19_3z_PD-1_28 T cells of the final product 12 days after activation. (K) PD-1 surface expression was higher on CAR^+ T cells compared to CAR^- T cells due to PD-1-CD28 fusion protein expression. Effector T cells = $CD62L^-/CD45RO^-/CD95^+$, effector memory T cells = $CD62L^-/CD45RO^+/CD95^+$, central memory T cells = $CD62L^+/CD45RO^+/CD95^+$, stem cell-like memory T cells = $CD62L^+/CD45RO^-/CD95^+$, naïve T cells = $CD62L^+/CD45RO^-/CD95^-$. N≥3 individual donors (all panels). Statistical significance was calculated using t test. E:T ratio = effector to target ratio.

Supplementary Figure 3: Transduction of cell lines and cytotoxicity of 1st generation PD-1-CD28 CARs. (A) K562 cells were transduced with CD19 or CD19 and PD-L1. Exemplary flow cytometry plots are shown proving comparable CD19 transduction rates. (B) Conventional 1st generation CAR T cells (19_3z) and 1st generation CAR T cells with fusion protein (19_3z_PD-1_28) were co-cultured with $CD19^+/PD\text{-}L1^+$ K562 cells. Cytotoxicity was analyzed 48 hours later. N≥3 individual donors (B).

Statistical significance was calculated using t test. UT = untransduced T cells, E:T ratio = effector to target ratio.

Supplementary Figure 4: Transduction rates of anti-CD22 CAR T cells with PD-1-CD28 fusion protein. (A) Transduction rate of anti-CD22 CARs with and without fusion protein was analyzed by flow cytometry 7 days after activation. (B) Exemplary flow cytometry plots showing strong correlation between CAR expression and PD-1 expression in PD-1-CD28 anti-CD22 CARs. N≥2 individual donors (A).

Supplementary Figure 5: Killing capacity after multiple antigen encounter. Target-cell killing throughout the multiple stimulation assay of T cells derived from 2nd donor is shown (see Figure 2A).

Supplementary Figure 6: Nalm-6/PD-L1 *in vivo* model. (A) *In vivo* characteristics of BCP-ALL with PD-L1 overexpression were analyzed. Survival curve of NSG mice injected with 1x10^5 conventional Nalm-6 cells or Nalm-6 cells with PD-L1 overexpression (n=5). Statistical significance was calculated using a Wilcoxon matched-pairs signed rank test. (B) Total radiance of NSG mice injected with conventional Nalm-6 cells and different CAR T cells (see Figure 2D). (C) Frequency of CD19$^+$ leukemia cells in the peripheral blood was analyzed 22 days after T-cell injection. Statistical significance was calculated using Log-rank (Mantel-Cox) test (A).

Supplementary Figure 1

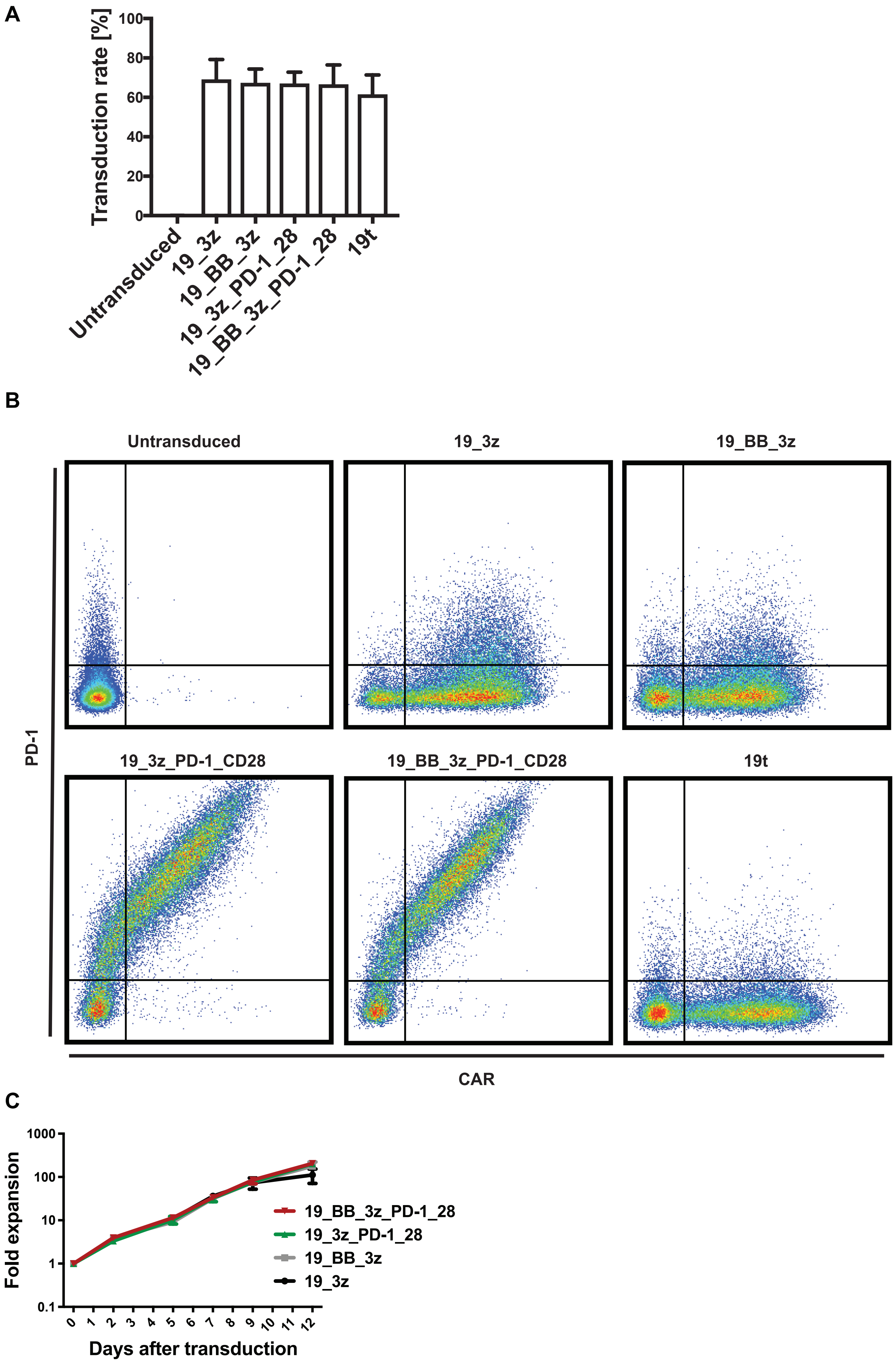

Supplementary Figure 2

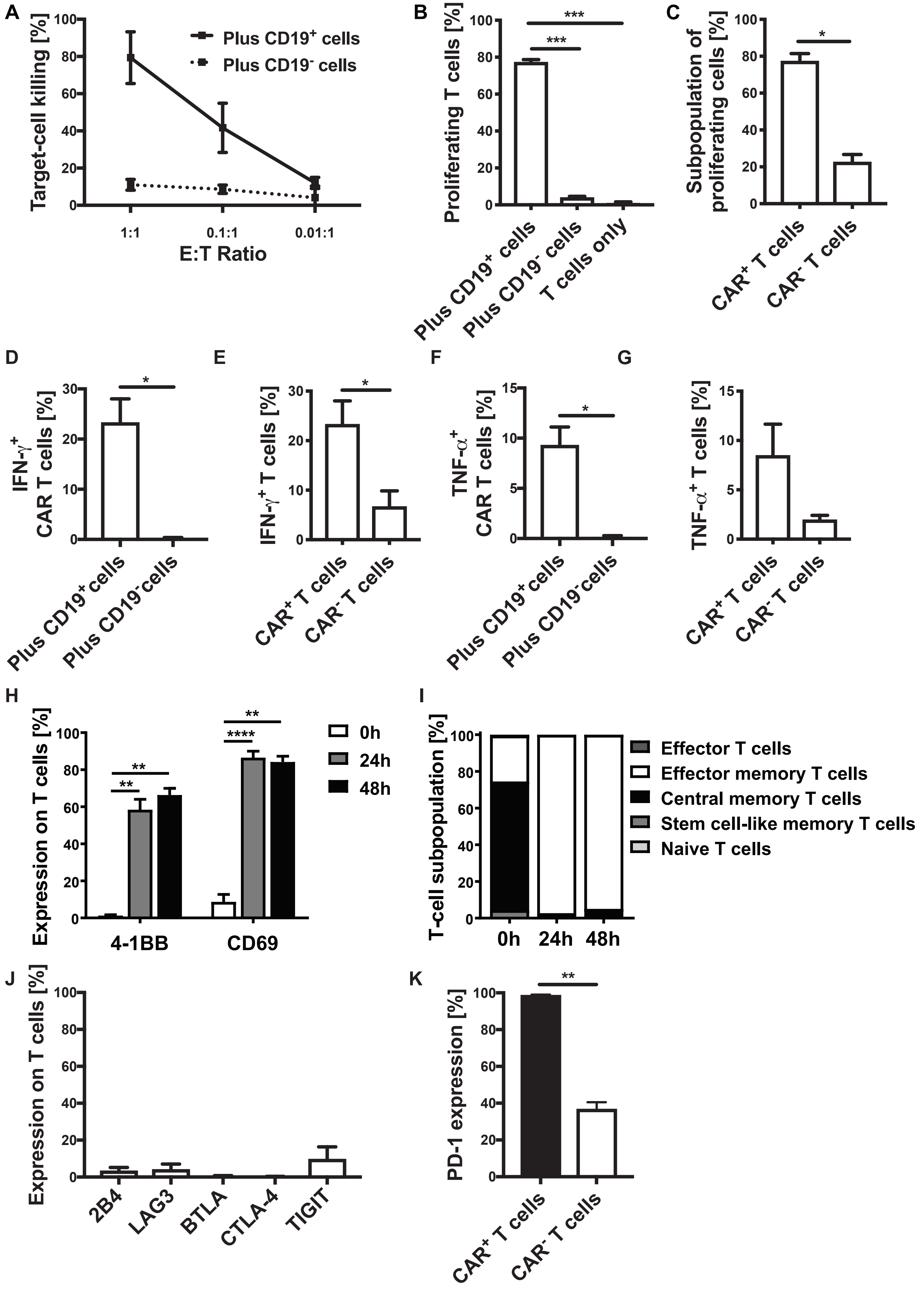

Supplementary Figure 3

A

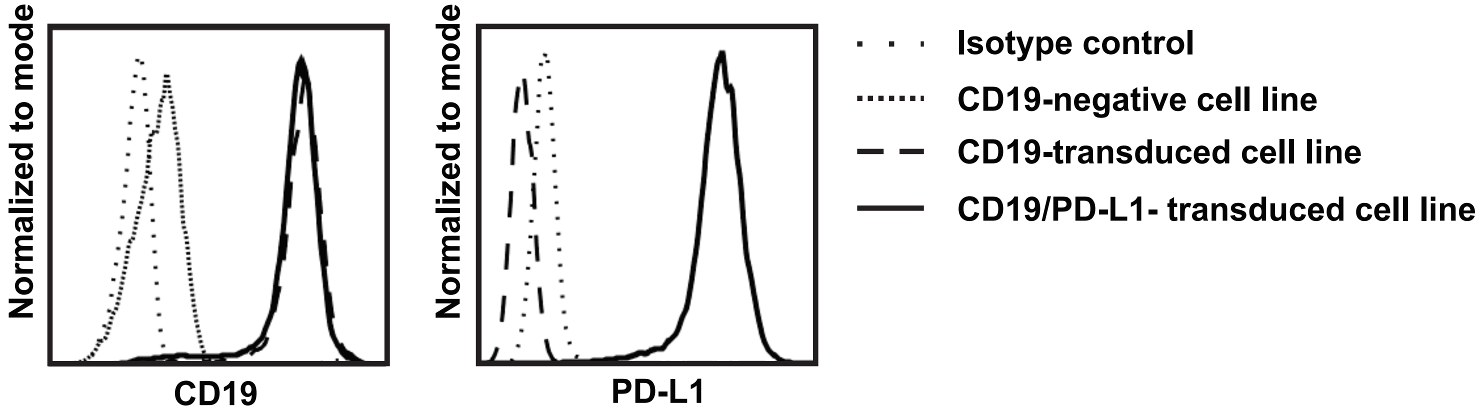

B

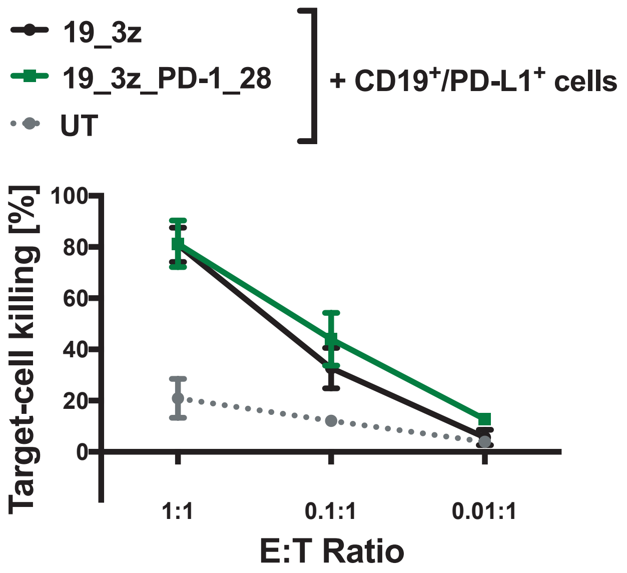

Supplementary Figure 4

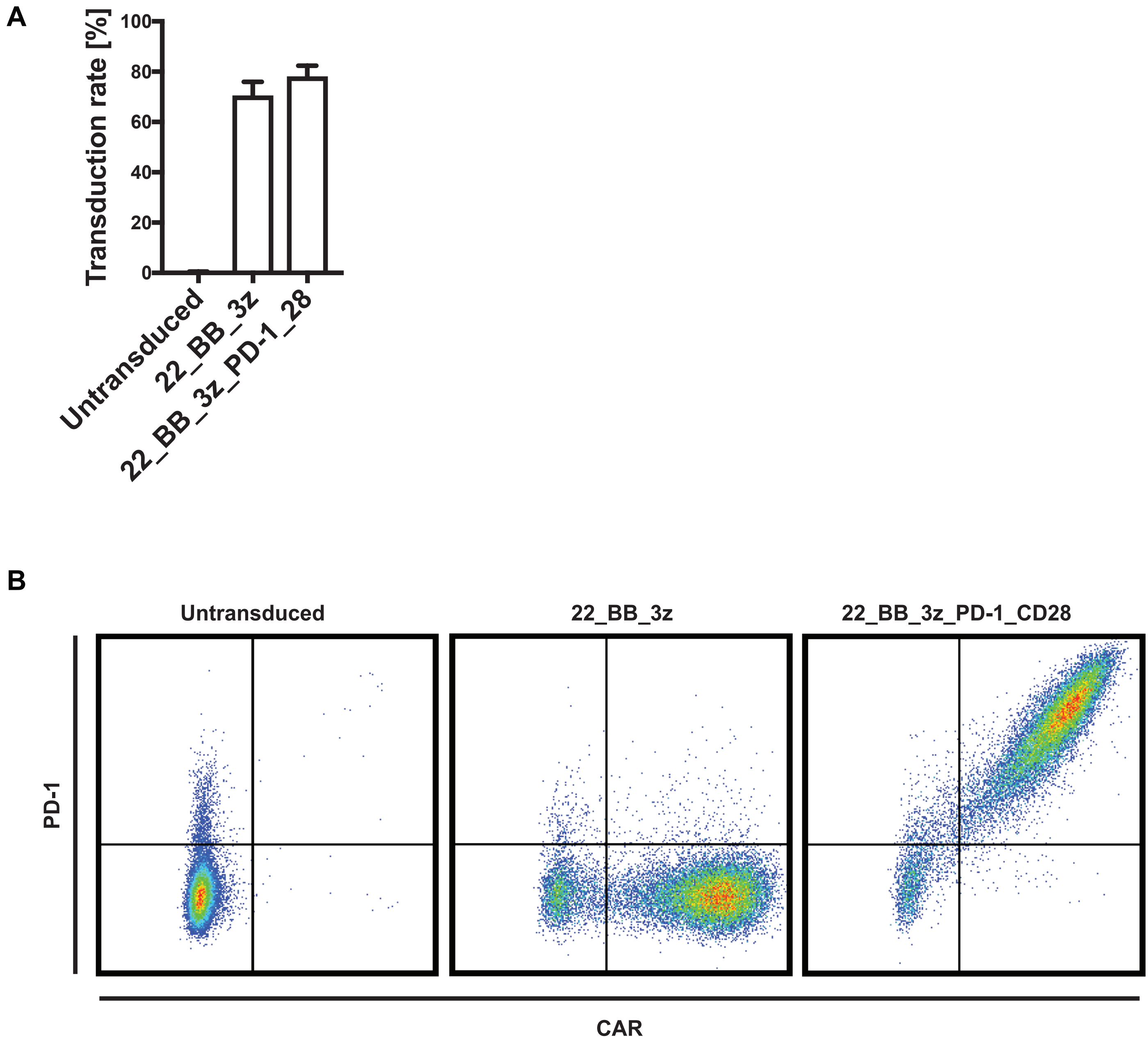

Supplementary Figure 5

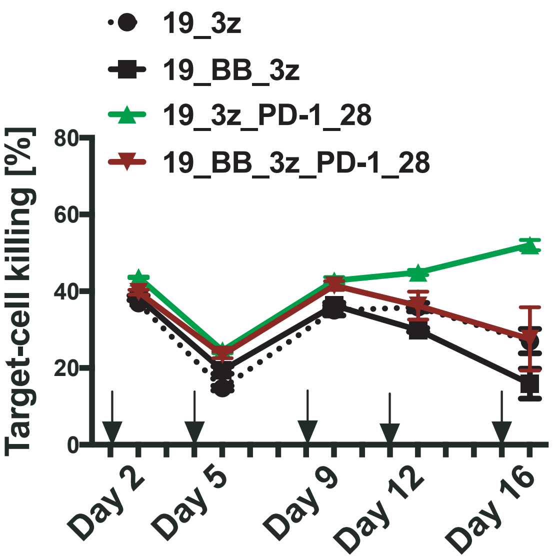

Supplementary Figure 6

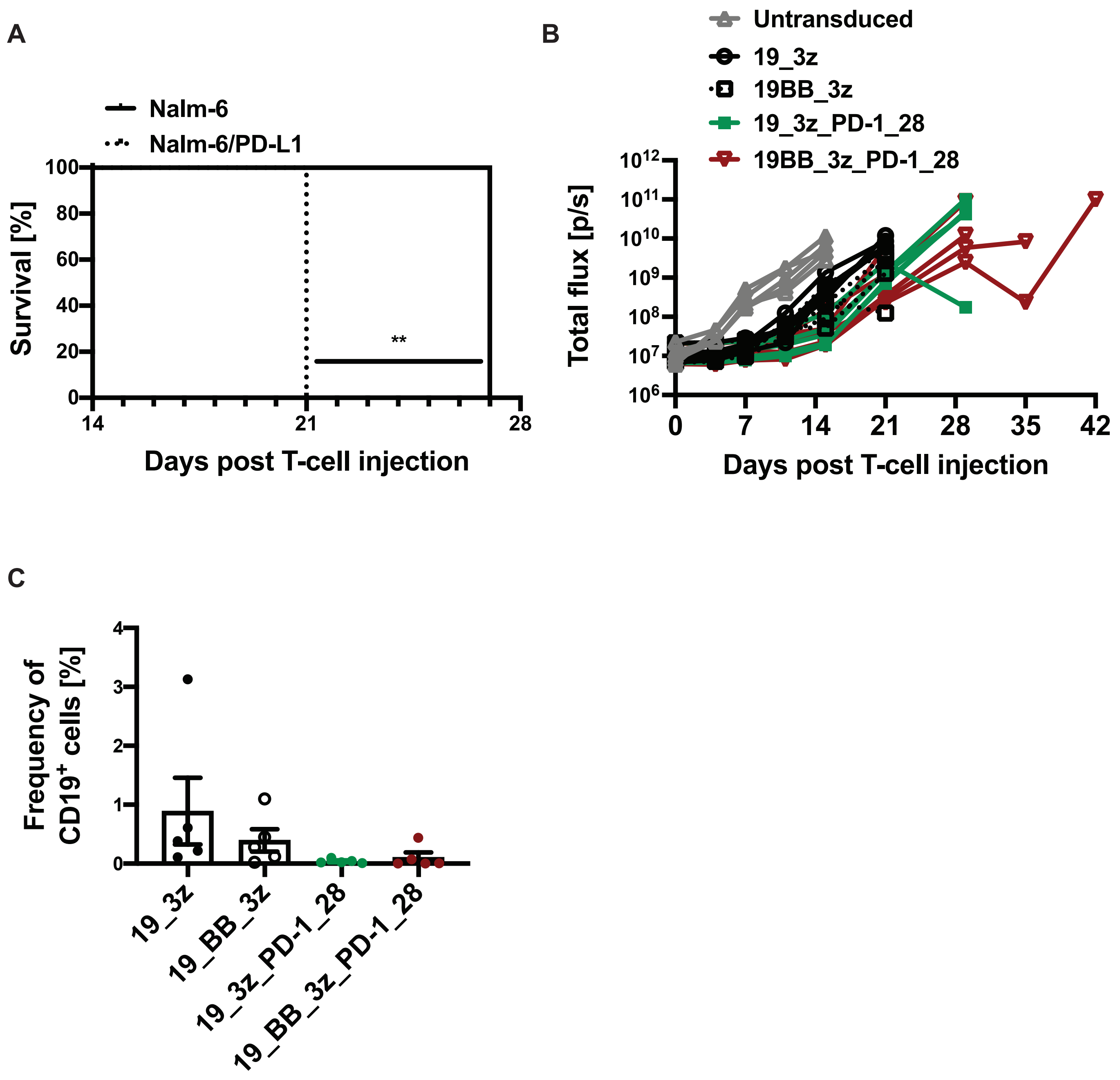

Supplement: Supplementary file 1 — Supplement [file 41408_2021_499_MOESM1_ESM.pdf]
